# The Effect of α-Olefin–Maleic Anhydride Copolymer on the Rheological and Crystalline Properties and Microcellular Foaming Behavior of Polyamide 6

**DOI:** 10.3390/polym15092056

**Published:** 2023-04-26

**Authors:** Shengnan Li, Tuanhui Jiang, Xiangbu Zeng, Nenggui Zhu, Chao Shen, Wei Gong, Chun Zhang, Li He

**Affiliations:** 1 National Engineering Research Center for Compounding and Modification of Polymer Materials, Guiyang 550016, China; lsn_gzyx@163.com (S.L.); zxbmold@163.com (X.Z.); zhung1013@163.com (N.Z.); qiubabe825@163.com (C.S.); 2 Institute of Materials and Construction, Guizhou Normal University, Guiyang 550025, China; gongw@gznu.edu.cn; 3 School of Materials and Metallurgical Engineering, Guizhou Institute of Technology, Guiyang 550025, China; zhangchun925@126.com

**Keywords:** polyamide 6, chain extender, rheological properties, foaming behavior, water absorption

## Abstract

The α-olefin–maleic anhydride copolymer DIA as a chain extender was used to modify polyamide 6 (PA6) during melt blending. The ability to modulate this modification for PA6 has been shown to be dependent on the effects of its content on the molecular weight distribution, rheological properties, crystalline properties, mechanical properties, and foaming behavior of foam samples. By increasing the DIA content, the viscoelasticity, water contact angle, and elongation at break improved as a result of a significant decrease in water absorption and melt flow rate. Compared with raw PA6, the modified PA6 presented a relatively wider molecular weight distribution. However, the crystallinity of modified PA6 samples decreased, the double melting peaks became one peak, and the α crystallites at 20.3° gradually disappeared with increasing DIA content. The morphologies of composite foams with different contents were analyzed using scanning electron microscopy. It was found that the cell size of different PA6 samples decreased from 160 μm to 83 μm and the cell density increased from 1.1 × 10^5^ cells/cm^3^ to 5.9 × 10^5^ cells/cm^3^ when the content of DIA increased from 0 wt% to 5 wt%. Meanwhile, the cell morphology obviously improved and the cell size distribution became narrow. Thus, a preparation technology based on foaming materials with excellent performance, such as better bubble quality and low water absorption, was developed for further research and application.

## 1. Introduction

Polyamide 6 (PA6), belonging to the aliphatic polyamide family, has been widely used in automotive and electronical fields due to its outstanding comprehensive properties [1,2]. Microcellular PA6 foams not only reduce product density and material costs but also have excellent energy absorption and anti-impact properties [3]. However, PA6 generally has a linear molecular structure containing amide groups in the main chain, and the polymer chain segment contains strong polar amide groups (-CO-NH-), making it easy to interact with water (hydrophilic) [4]. Moreover, it has strong hydroscopicity and low melt viscosity, which limits its foaming ability and then hinders application [5,6]. The use of a chain extender in PA6 can effectively overcome these limitations.

Generally, many modified strategies, such as fiber reinforcing [7], inorganic filling [8], chain extension [9,10], cross-linking [11], and long-chain branching [12,13], have been used to enhance the melt strength or melt elasticity of PA6. Among these, the methods of fiber reinforcing and inorganic filling are relatively mature technologies. Recently, there has been renewed interest in chain extension and long-chain branching [14]. By modifying the molecular chain structure and increasing the molecular weight, long-chain branching is selected as a particularly efficient way to improve foamability. Consequently, a variety of chain extenders (CEs), including epoxides [11,15], oxazolines [16], and maleic-anhydride-functionalized polymers (MA-f-pol) [17], have been used. Han et al. [18] used a CE containing 10 wt% multifunctional epoxides to obtain a high melt strength, which was accompanied by a decrease in the cell size and enhanced foaming ability and cell density of PA6 due to the formation of induced crystals. Xu et al. [13] prepared long-chain branched (LCB) PA6 with varying degrees of long-chain branching using a multifunctional epoxide oligomeric agent as a chain extender and maleic-anhydride-grafted polypropylene as a branching agent. It was concluded that reaction extrusion could be used to produce LCB PA6 through an incredibly high degree of long-chain branching, high melt strength, and toughness properties. They also used MAH-g-PP, a co-chain extender, and ADR (styrene–acrylic multifunctional epoxide oligomeric agent), resulting in high-molecular-mass branching PA6 with an LCB semantic network [12]. It was observed that LCB PA6 with longer branches presented a wider molar mass distribution and higher molecular weight. Moreover, LCB PA6 with a longer branch length and higher melt strength was also able to achieve wider foaming temperature ranges. In summary, although many methods have been used to improve modified PA6′s molecular weight and melt strength, there are few references on the modifying agent α-olefin–maleic anhydride copolymer [19].

So far, there is little information on modifying PA6 with the α-olefin–maleic anhydride copolymer DIA as a CE. In this study, we attempted to create comb-like structured PA6 foams via chemical microcellular injection molding technology using a maleic-anhydride-containing polymer as a CE, in which the α-olefin–maleic anhydride copolymer possesses excellent recyclability and viscosity stability at high temperatures. The contents of DIA were controlled to adjust the structure and properties of the modified PA6. The effects of the DIA content on the rheological properties, crystalline properties, foaming behavior, and mechanical properties of PA6 were systematically investigated.

## 2. Experiment

### 2.1. Materials

PA6 was provided by Guangdong Xinhui Meida Nylon Co., Ltd. (Jiangmen, Guangdong, China), with a melt index of 24 g/min (235 °C, 2.16 kg), a density of 1.14 g/cm^3^, and a relative viscosity of 2.58. The α-olefin–maleic anhydride copolymer DIA was used as a CE and was provided by Mitsubishi Chemical Corporation (Japan) under its trade name Diacarna 30M (hereinafter referred to as DIA). The chemical foaming masterbatch (trade name: EE25C; gas production volume: 35 mL/g; decomposition temperature: 160–200 °C) was provided by Eiwa Fine Chemical Industry Co., Ltd. (Changshu, China).

### 2.2. Sample Preparation

To reduce the impact of moisture, raw PA6 was dried in an oven at 105 °C for 5 h prior to melting and mixing. In a twin-screw extruder (CTE35, Coperion Keya (Nanjing) Machinery Co., Ltd., Nanjing, China), PA6 and DIA were melt-blended to prepare modified PA6 samples at various mixture ratios (99/1, 98/2, 97/3, 95/5, and 92/8 by weight). The screw diameter was 35.6 mm, and the length–diameter ratio was 36. The corresponding sample names were raw PA6, PA6–DIA1%, PA6–DIA2%, PA6–DIA3%, PA6–DIA5%, and PA6–DIA8%, respectively. The screw speed was set to 250 rpm, and the temperature varied from 190 to 200, 210, 220, 225, 225, 230, 230, and 220 °C from the hopper to the die. All the samples of raw and modified PA6 were dried once more at 105 °C for 5 h before the foaming process.

**Figure 1:**
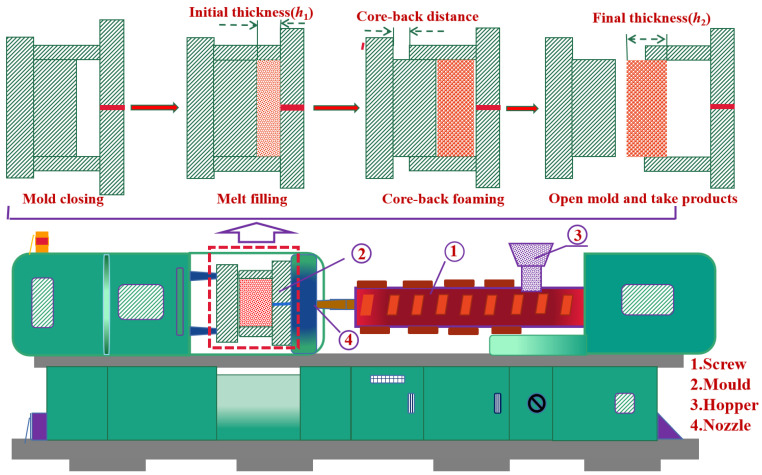
Schematic diagram of the core-back foaming process and the injection molding device.

The dried PA6 granules and foaming masterbatch (2 wt%) were mixed and then filled into an injection molding machine (EMI 120-V, Zhen de Plastic Machinery Co., Ltd., Guangdong, China) for injection molding. The barrel’s temperature differential from the hopper to the nozzle was 220 to 230 to 240 °C. Core-back foaming technology was used to prepare foaming samples, and the specimens were dumb-bell-shaped types. A schematic diagram of the core-back foaming process and the injection molding device are shown in Figure 1.

### 2.3. Characterizations

#### 2.3.1. Fourier Transform Infrared Spectra (FTIR)

Fourier transform infrared spectroscopy (Nicolet IS50, Thermo Fisher Scientific, Waltham, MA, USA) was used to study the interaction between the raw PA6 terminal group and DIA functional groups. Different PA6 samples were measured using 32 scans at a resolution of 4 cm^−1^. Each spectrum was obtained at 4000–400 cm^−1^.

#### 2.3.2. Gel Permeation Chromatography (GPC)

The weight-averaged molecular weights (Mw), number-averaged molecular weight (Mn), and polydispersity index (PDI) of different PA6 samples were obtained using GPC measurements (PL-GPC220, Agilent Technologies Inc., Santa Clara, CA, USA) at 40 °C with hexafluoroisopropanol (HFIP) as the eluent at a 1 mL/min flow rate.

#### 2.3.3. Rheological Behavior

The dynamic shear rheological characteristics of raw and modified PA6 samples were measured using a rotational rheometer (HAAKE MARS60, Thermo Fisher Scientific, Waltham, MA, USA) with parallel plate geometry. The test specimens were round tablets with a diameter and thickness of 20 mm and 1.6 mm, respectively. Next, frequency sweep tests were conducted at 225 °C to obtain the dynamic characteristics of all samples in a frequency range from 0.05 to 100 Hz for a predetermined strain amplitude of 2% (to ensure a linear viscoelasticity region in the tested polymer melt). For each sample group, at least two parallel experiments were conducted to guarantee the accuracy of the results.

#### 2.3.4. Melt Flow Rate (MFR)

A melt flow rate meter (MTS/SANS ZRZ2452, MTS Systems (China) Co., Ltd., Shanghai, China) was used to determine the MFR of PA6 samples. The timer was set to change the cutoff time for a time interval of 10 s. The experimental temperature and load mass were 235 °C and 2.16 kg, respectively.

#### 2.3.5. Differential Scanning Calorimetry (DSC)

Using TA Instrument (DSC-25, TA Instruments, New Castle, DE, USA), the melting and crystallization behaviors of different PA6 samples were studied. In nitrogen gas ambience, the different PA6 samples were first heated to 250 °C and maintained at that temperature for 3 min to eliminate thermal history, then cooled to 40 °C at a rate of 5 °C/min, and finally heated up to 250 °C at the same rate. The relative crystallinity (*Xc*) of different PA6 samples was calculated using the following Equation (1) [5]:

(1) $$X_{c}=\frac{\Delta H_{m}}{\Delta H_{m}^{0}}\times 100\%$$

where $\Delta H_{m}$ and $\Delta H_{m}^{0}$ were the experimental melting enthalpy of PA6 samples and the complete melting enthalpy of crystallized PA6 (190.8 J/g), respectively.

#### 2.3.6. X-ray Diffraction (XRD)

XRD analysis (ARL Equinox 3000, Thermo Fisher Scientific, Waltham, MA, USA) was carried out with a Cu Kα (1.54 Å) radiation source generated at a voltage of 40 kV and a current of 40 mA. The X-ray diffraction curve was recorded at a scan range of 3°–100°.

#### 2.3.7. Hydrophilic and Water Absorption Test

An optical contact-angle-measuring device (DSA25, Kruess Scientific Instrument Co., Ltd., Hamburg, Germany) was used to characterize the water contact angle (CA) of the specimens. Composite specimens were created with dimensions of 40 mm × 10 mm × 4 mm, and then, the specimens were dried at 80 °C for 10 h for use. Deionized water was used as the liquid phase, and a CA tester was used to test the CA between the specimens and water. At least three samples were measured for each group, and the average value was considered.

Specimens with dimensions of 40 mm × 10 mm × 4 mm were dried in a drying oven at 80 °C for 12 h and then were immersed in distilled water at room temperature for 48 h. Each specimen’s change in mass, or the difference between its starting mass and that after exposure to water, was used to calculate the content of water that it absorbed. The change in mass was expressed as a percentage of the initial mass. The appropriate calculation (Equation (2)) was used to determine the percentage change in mass, *C*, related to the beginning mass for each test specimen:

(2) $$C=\frac{m_{2}-m_{1}}{m_{1}}\times 100\%$$

where C is the percentage of mass change before and after water absorption; $m_{1}$ is the weight of the specimen after drying, in mg; and $m_{2}$ is the weight of the specimen after soaking, in mg.

#### 2.3.8. Scanning Electron Microscopy (SEM)

The microstructures of PA6 foams were observed with SEM (KYKY-EM6000, KYKY Technology Co., Ltd., Beijing, China). Different PA6 foams were submerged in liquid nitrogen for 4 h before being immediately broken up in air. Next, a small layer of Au was applied to the cracked surfaces of different PA6 foams before observation. To determine the average cell size and cell density, SEM micrographs were examined using Nano measure software. Equation (3) was used to obtain the cell density (*N*), which is the number of cells per cubic centimeter of solid polymer [12]:

(3) $$N={\left(\frac{n}{A}\right)}^{\frac{3}{2}}\cdot \frac{\rho_{0}}{\rho_{f}}$$

where *n* is the number of cells in the statistical area, *A* (cm^2^) is the statistical area of the micrograph, $\rho_{0}$ is the density of the solid samples, and $\rho_{f}$ is the density of the foamed samples.

#### 2.3.9. Mechanical Properties

In accordance with GB/T 1040-2006 standards, tensile tests were carried out to assess the mechanical performance of the injection-molded PA6 samples. The tensile tests were conducted on a universal electronic testing apparatus (SANS CMT6104, MTS Systems (China) Co., Ltd., Shanghai, China). The head speed rates were maintained at 50 mm/min during the tensile tests. At least five samples from each group were measured, and the average result was used for discussion.

## 3. Results and Discussion

### 3.1. Chain Extension Characterization

**Figure 2:**
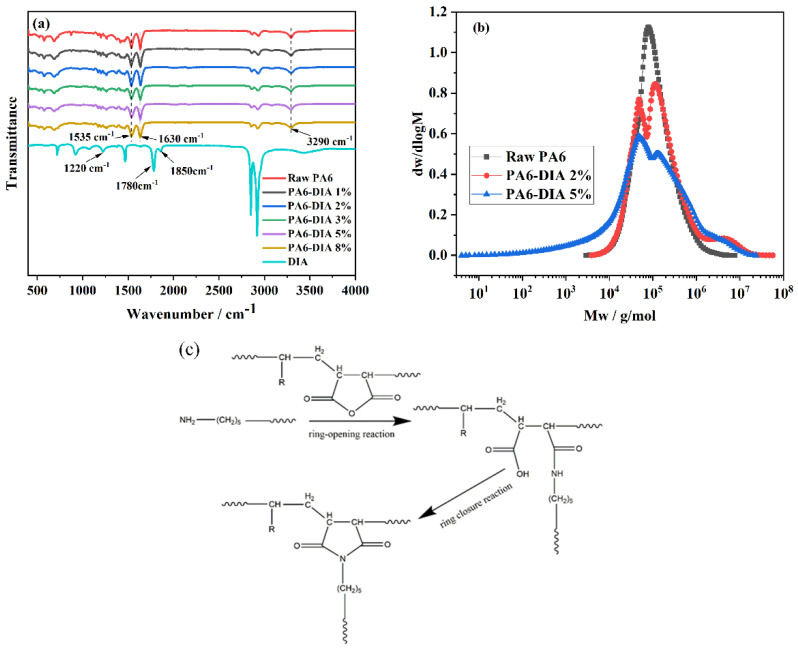
(**a**) FTIR spectra of different PA6 samples and DIA, (**b**) molecular weight distributions of raw PA6, PA6–DIA2%, and PA6–DIA5% samples, and (**c**) proposed mechanism of the reaction between PA6 and DIA.

Figure 2a shows the FTIR spectra of DIA and different PA6 samples. The bands at 1630 cm^−1^ (C=O stretching vibration) and 1535 cm^−1^ (N–H in-plane bending vibration) in the FTIR spectra of raw PA6 correspond to the amide group in PA6. Meanwhile, the amine group’s stretching vibration in the PA6 samples was responsible for the band at 3290 cm^−1^, and the out-of-plane bending vibration of primary amine (N–H_2_) was responsible for the band at 690 cm^−1^. In the DIA spectrum, there were two peaks at 1850 cm^−1^ and 1780 cm^−1^, which contributed to the characteristic stretching vibrations of the carbonyl group of MAH, and the band at 1220 cm^−1^ contributed to the C–O stretching of the anhydride. After the melt mixing process, the peaks of the anhydride’s carbonyl group C=O and C–O stretching disappeared from the spectrum of the modified PA6 samples. Meanwhile, the bands of the amide group (the bands at 1630 cm^−1^ and 1530 cm^−1^) became weaker, and N–H_2_ bending vibration appeared in the modified PA6 samples, indicating the occurrence of a chain extension reaction between PA6 and DIA.

A GPC test was performed to further confirm these results. One of the crucial factors affecting the characteristics of polymeric materials, including viscosity, mechanical strength, crystallization behavior, and morphological phase, is the polymer’s molecular weight distribution (MWD). For instance, a small fraction of extremely long molecules significantly increases the melt viscosity, whereas a small number of short molecules significantly decreases the breaking strength [20]. Therefore, GPC tests were conducted to figure out the effects of chain extension on the MWD of the modified PA6 samples. Figure 2b shows the MWD of the raw and modified PA6 samples, and Table 1 summarizes additional specific information about the number-averaged molecular weight (Mn), weight-averaged molecular weight (Mw), and polydispersity index (PDI). Because of the chain extension between PA6 and DIA, the modified PA6 curves showed a substantially broader MWD than the raw PA6 curve. Especially for the 5 wt% sample, the MWD was wider, suggesting that the size of the polymer chain is even more uneven.

As is well known, low-molecular-weight substances make a significant contribution to Mn (because a lower molecular weight leads to more molecular data). On the contrary, Mw is more influenced by the macromolecules in materials (because the larger the molecular weight, the heavier the individual molecule) [21]. The PDI, equal to the ratio of Mw to Mn (Mw/Mn), represents the overall distribution of macromolecules and small molecules (the larger the PDI, the wider the MWD). The PDI is not a thorough representation of the precise molar quantities of each chain size, and it merely describes the relative breadth of molar masses. With an increase in the DIA content, we saw a trend in the peak molecular weight (Mp) to high molecular mass.

From Table 1, we observed that the Mn of modified PA6 samples decreased. However, the Mw of modified PA6 samples significantly increased to as much as two times that of raw PA6, indicating there are more macromolecular substances. The Mw of PA6 samples increased from 1.3 × 10^5^ g/mol to 2.7 × 10^5^ g/mol and 2.5 × 10^5^ g/mol when the content of DIA increased from 0 wt% to 2 wt% and 5 wt%, respectively. In addition, the PDI of the modified PA6 samples was higher than that of raw PA6. This indicated that long-chain branched PA6 was produced as a result of anhydride groups reacting with amino end groups. The population of high-molecular-weight chains increased due to the presence of branched structures [15]. Subsequently, rheological testing of different PA6 samples was used to confirm the relationship between the MWD and complex viscosity.

Figure 2c illustrates the potential molecular topological structure of final products and the proposed mechanisms of chain-branching reactions. In reality, the cyclic anhydride reactions depicted in Figure 2c require two steps: ring opening to amic acid and then ring closing by removing the water. In the first step, the reactive hydrogen of the amino end group in PA6 attacked the anhydride’s carbon–oxygen bond, resulting in the ring-opening reaction of the anhydride group. Subsequently, the opened rings reacted with the amino end group of the PA6 chains to make chain-extended products. In the second step, primary amines could quickly react with the unstable carboxylic group to form a stable cyclic imide structure after a ring-closing reaction. Considering the anhydride groups and the side chain (which is long-chain hydrocarbons) in DIA, the products could be branched in design. With different DIA concentrations, a comb-like structure was obtained in the modified PA6 samples.

### 3.2. Rheological Properties

**Figure 3:**
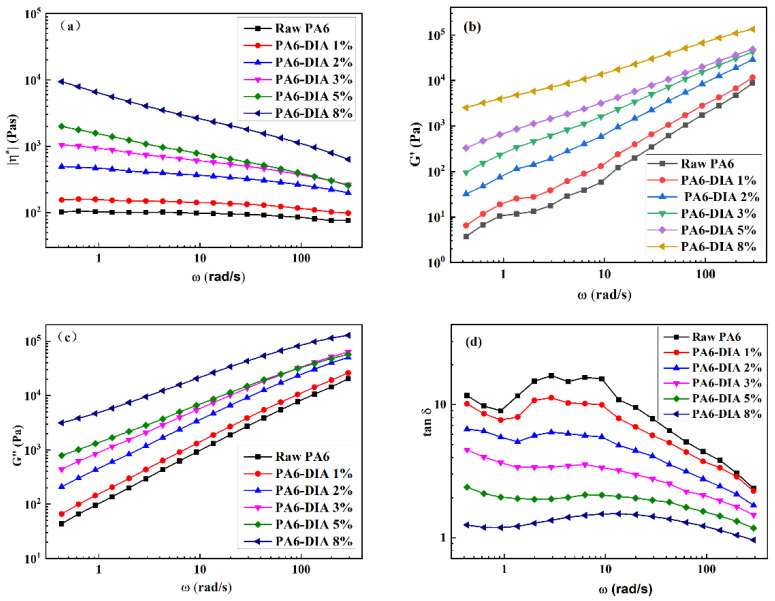
Dynamic rheological properties of different PA6 samples: (**a**) η*, (**b**) G’, (**c**) G”, and (**d**) tanδ.

The rheological properties of polymers are thought to be closely related to their melt strength and are a key determinant in influencing the polymers’ foamability. Figure 3 exhibits the dependence of the complex viscosity η*, the storage modulus G’, the loss modulus G”, and the loss tangent tanδ on the angular frequency ω of different PA6 samples. In Figure 3a, raw PA6 showed a low η* and a broad Newtonian plateau. With an increase in the CE amount, the samples exhibited non-Newtonian behavior in the studied frequency range with a shear-thinning phenomenon and had a much higher η* [22]. This is explained by an increase in PA6′s molecular weight (Figure 2b and Table 1). The increasing trend of η* indicates that the materials’ chain extension potential for different PA6 samples during extrusion increased. The structural variations can explain the improvement of chain-extended products with DIA. Chain-extended products with DIA showed larger molecular weights than raw PA6, which consists of a linear molecular architecture (Figure 2c). This is due to the comb-like structure in the consequent materials generated from the reaction of PA6 with DIA [23]. However, η* changed a lot with the addition of DIA beyond 5 wt%. There were two main reasons for this. On the one hand, the comb-like structure in the chain-extended PA6 caused a significant number of molecular entanglements, and when the shear rate increased, the molecular chains in modified PA6 became untangled [22]. On the other hand, the higher the proportion of low-molar-mass chains in these samples, the lower the complex viscosity at higher shear frequencies [24].

The melt elasticity, viscosity, and foaming behavior of polymers were also significantly influenced by the G’ and G” of those materials. The main element regulating cellular morphology during the growth of cells was melt elasticity. High melt elasticity could assist the growth of bubbles and stop them from collapsing, resulting in foams with a regular cell structure. As shown in Figure 3b,c, the G’ and G” of the samples had a similar change trend. Both G’ and G” increased with DIA addition, indicating that chain extension improves the corresponding melt elasticity. In turn, the increase in melt elasticity and strength improved the foamability of PA6 samples.

The loss tangent tanδ is defined as a ratio (G”/G’), which is also known as the ratio of viscous-to-elastic contribution (damping behavior) at a given angular frequency. Throughout the frequency range, the tanδ values of the modified PA6 samples were lower than those of raw PA6, as shown in Figure 3d. This indicated that the modified PA6 samples may have acquired a high melt elasticity and a fast elastic response. The elastic response became faster, the viscous dissipation significantly decreased, and the foamability improved. This should be attributed to the creation of branching structures, which increased the amount of entanglement between PA6 molecular chains [25].

**Figure 4:**
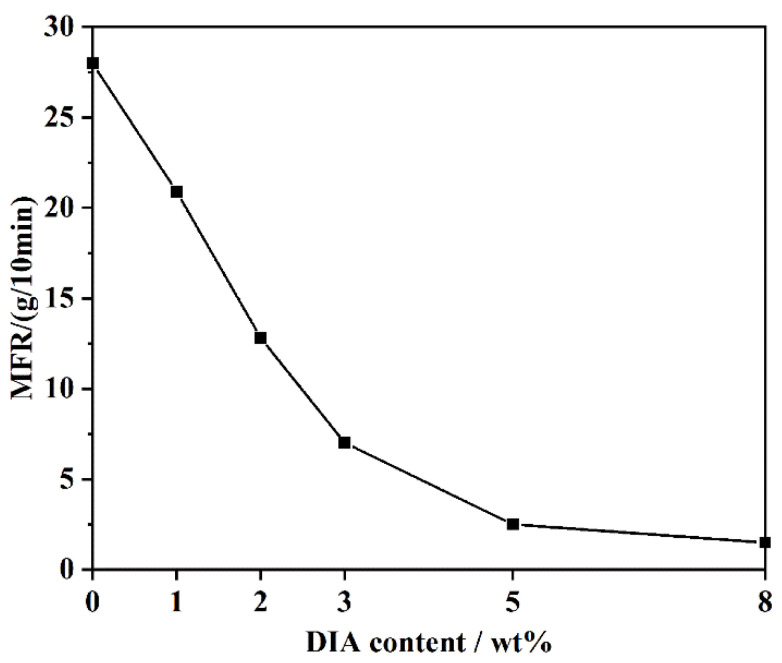
Melt flow rates of different PA6 samples.

The MFRs of PA6 samples were measured for further evaluating the effect of DIA on flowability. As shown in Figure 4, the existence of a CE deteriorated the flowability of PA6 melt, which indirectly indicated that chain extension occurred between PA6 and DIA in the melt-blending process. Furthermore, the MFR value decreased as the CE content increased, which indicated that the incorporation of DIA increased the viscosity of modified PA6. With the DIA content increasing from 0 wt% to 3 wt%, the MFR decreased quickly from 28 to 7 g/10 min. However, the MFR fell slowly down to 2.5 and 1.5 g/10 min when the DIA content was 5 wt% and 8 wt%, respectively. The reduced flowability is derived from the long-chain branching structure, which produced entanglement networks. This structure forcefully affected the polymer’s rheological characteristics. The results agreed well with the rheological measurement.

### 3.3. Crystallization Properties and Crystal Structure

**Figure 5:**
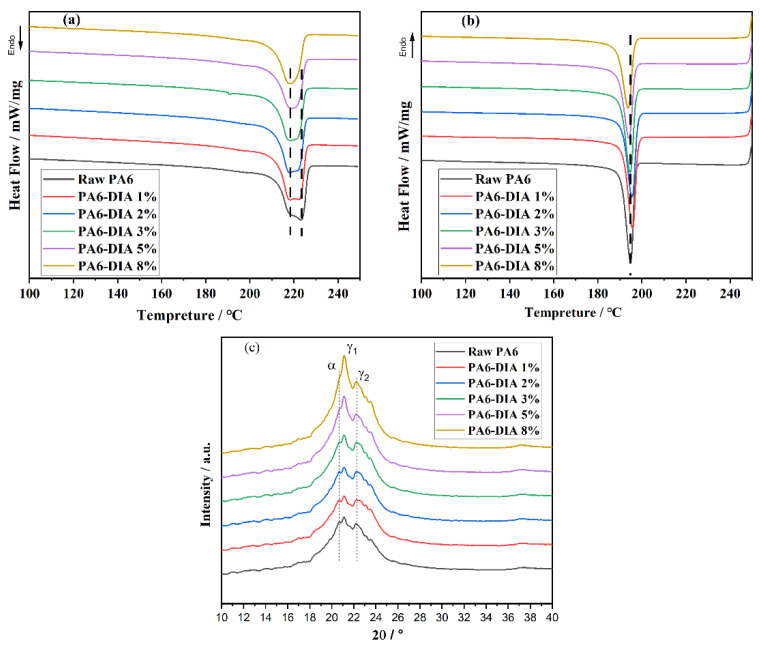
(**a**) DSC heating melting curve of different PA6 samples, (**b**) DSC cooling crystallization curves of different PA6 samples, and (**c**) XRD patterns of different PA6 samples.

To confirm the effects of the DIA content on the thermal properties and crystal structure of PA6, DSC and XRD were used to measure the crystallization and melting properties of PA6 samples. Figure 5a,b and Table 2 present the DSC curves of PA6 samples and the corresponding thermal property parameters, respectively. As reported by other researchers, in the major melting region (i.e., in the temperature range of 205–230 °C) of PA6 samples, there were two melting peaks, T_m,1_ (at about 222 °C) and T_m,2_ (at about 218 °C), which corresponded to the melting events of α-form and γ-form crystals, respectively [26]. When the DIA content was more than 3 wt%, the double melting peaks disappeared and merged into one peak at about 218 °C. It was also found that the crystallinity (Xc) of modified PA6 was lower than that of raw PA6, which indicated that the branching structures were formed after the DIA reduced the crystallization ability of PA6 [18]. The density of the polymer chains’ intermolecular hydrogen bonds would have decreased due to the branching structure [27]. In addition, macromolecules with side groups decreased the cross section of molecular chains and increased their flexibility, partially hindered the movement of chain segment, and influenced how the polymer chains were arranged in a crystalline structure. When the content of DIA was 5 wt% and 8 wt%, Xc reached the lowest value. This is a clear sign that the chain extension reaction partially prevents PA6 from crystallizing. The increase in the molecular weight of chain-extended products (Figure 2b and Table 1) caused a decrease in the crystallinity and made it more difficult for the polymer chain to be arranged in a crystalline structure. Similar observations regarding the effect of a CE on the crystallization behavior of polymers have been previously reported by Tuna et al. [9] and Lu et al. [16] in PA6 chain extension studies. According to those studies, the decrease in crystallinity was attributed to three factors: the introduction of ester-amide groups to the main chain, the production of free caprolactam by-products, and an increase in the molecular weight of PA6 [23]. In raw PA6, the polymer chains were sufficiently mobile to form crystals. In modified PA6 samples, the greater molecular weight of chain-extended samples resulted in an insufficient time for the arrangement of polymer chains. In addition, the introduction of a cyclic imide group in the PA6 chain disrupted the chain regularity and created steric hindrance that reduced the number of PA6 chains that could crystallize [28].

PA6 is a semicrystalline polymer with a rather complex crystalline structure. Frequently observed crystalline forms are known as α and γ crystallites. The α crystallites are the most stable phase, and the γ crystallites are the unstable phase. As shown in Figure 5c, both crystallites coexisted in the different PA6 samples. The pronounced peaks at 21.2° and 22.3° represented γ crystallites, while the peak at 20.3°could be assigned to α crystallites. Another observation is that the intensity of the phases depended on the content of DIA. When the content of DIA was less than 3 wt%, the coexistence of α and γ crystallites was clearly observed. When the content of DIA was more than 3 wt%, the characteristic peak at 20.3° gradually disappeared, while the intensity of characteristic peaks at 21.2° became stronger with increasing DIA content. The γ crystallites gradually became a dominant population at the expense of the α crystallites. The results are quite consistent with the DSC results presented in Figure 5a,b.

### 3.4. Water Absorbency

**Figure 6:**
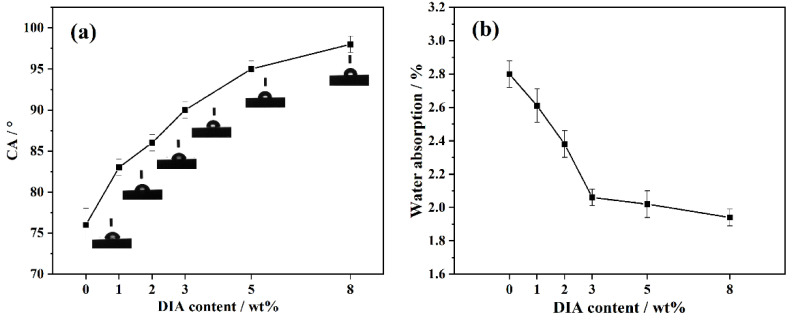
(**a**) Water CA values and (**b**) water absorption of different PA6 samples.

The side-chain R (long-chain hydrocarbons) in the α-olefin–maleic anhydride copolymer has an affinity for hydrophobic substances, which can induce a high water CA and low water absorption. Figure 6 shows the shape of water droplets on the surface of different PA6 samples and water absorption. The spreading effect of water droplets was different for different PA6 samples. The spherical shape became more complete with increasing DIA content. In contrast, the wettability was worse.

As can be seen in Figure 6a, the CA of the raw PA6 sample was 76°, indicating a hydrophilic surface. With increasing DIA content, the CA of the composites became larger. It was also found that the CA values of the samples were less than 90° when the content of DIA was less than 3 wt%. However, when the content of DIA was more than 3 wt%, the CA values of the samples increased, showing that the samples’ hydrophilicity decreased. This is because the inherent hydrophobic group of DIA worsened the infiltration of water into the composites, so the rate of water entering the material gap slowed down and the water absorption (as shown in Figure 6b) of the material decreased at the same time.

### 3.5. Foaming Performance of Different PA6 Samples

**Figure 7:**
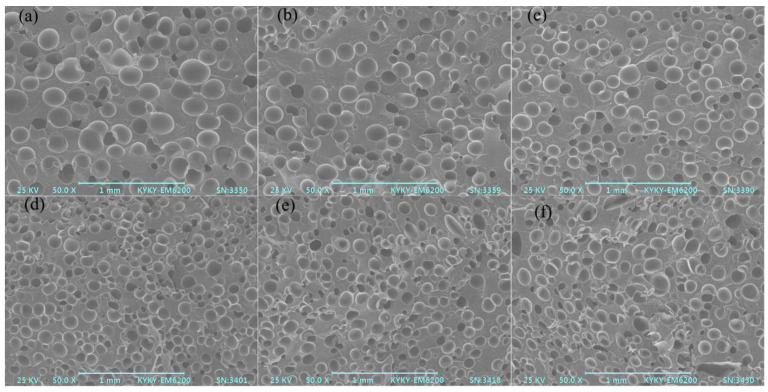

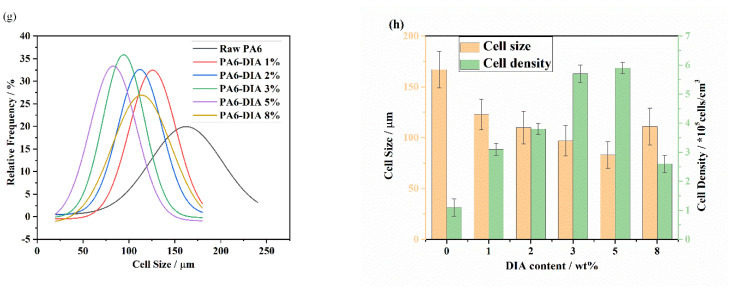
Microcellular morphology of different PA6 foams and DIA mass fractions: (**a**) 0 wt%, (**b**) 1 wt%, (**c**) 2 wt%, (**d**) 3 wt%, (**e**) 5 wt%, and (**f**) 8 wt%. (**g**) Cell size distribution of different PA6 foams and (**h**) variation of average cell diameter and cell density of different PA6 foams.

The cellular morphology, cell size distribution, corresponding average cell diameter, and foam expansion ratio, as well as cell density, of different PA foams are displayed in Figure 7. It was discovered that raw PA6′s cells showed significant rupture in the cellular morphology, with an average cell size of roughly 160 μm, a cell density of roughly 1.1 × 10^5^ cells/cm^3^, and a broader cell size distribution. The reason was that the melt strength was not enough to sustain cell growth.

After the addition of DIA, a smaller cell size and more uniform foam morphology were obtained. At the same time, the density of cells increased and the cell size distribution became narrow, especially when the amount of DIA added was 5 wt%. There are two reasons for this. First, rheological characteristics have a direct impact on the growth of cells and the stabilization of the cell structure during the foaming process. Many branching structures were generated, and the melt strength improved. The improved melt elasticity prevented cell rupture and mergence during the foaming process and hence improved foamability. Second, rapid crystallization, which stiffens the polymer matrix during the foaming process, may slow the active cell growth and harden the cell structure more quickly [26]. With a branched structure, the crystallization rate decreased due to the bad stereoregularity of the molecular chains and large steric hindrance of the substituents. Moreover, as the content of DIA increased, Xc decreased (Figure 5b and Table 2). At the stage of cell nucleation, premature crystallization of the melt would reduce the concentration of the blowing agent for nucleation, reduce the nucleation rate, lead to a decrease in the initial amount of bubble nucleation, and then affect the final bubble density. At the stage of bubble growth and stability, the crystallization behavior had a significant effect on the diffusion and solubility of gas in the melt. Too much crystallinity could reduce the diffusion and solubility of gas in the melt and affect the cell density and diameter. Therefore, premature crystallization of the melt could make the dissolved bubble quantities and the bubble growth power insufficient, causing a low cell density and large diameter. However, with the DIA content ranging from 5 wt% to 8 wt%, the cell density of foams decreased from 5.9 × 10^5^ cells/cm^3^ to 2.6 × 10^5^ cells/cm^3^, the cell size of foams increased from 83 μm to 111 μm, and the cell size distribution became wider. It was possible to infer from the rheological performance (Figure 3) that melt elasticity played a major role in the variation when the content of DIA was less than 5 wt%, while the melt viscosity reduced and the cells merged, leading to cell deformation, rupture, and coalescence when the DIA content was beyond 5 wt%.

### 3.6. Mechanical Property

**Figure 8:**
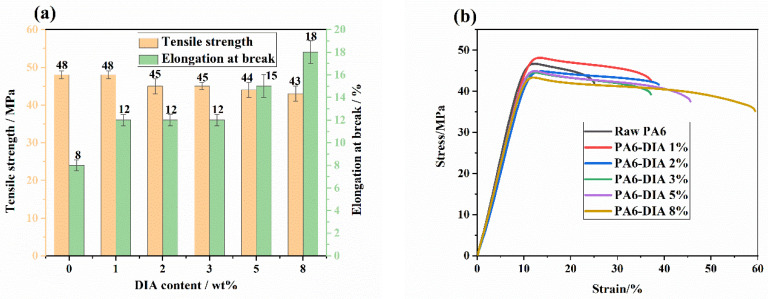
(**a**) Tensile strength, elongation at break of different PA6 samples, and (**b**) representative stress–strain curves of different PA6 samples.

Tensile tests were carried out to determine the influence of the CE content on tensile properties. As shown in Figure 8a, the DIA content can affect the tensile strength and elongation at break of PA6. With an increase in the content of DIA, the tensile strength slightly declined in contrast to the trend in elongation at break. The elongation at break of the chain-extended PA6 notably improved compared with that of raw PA6, and the 8 wt% PA6 sample had the highest value, which was approximately 2 times higher than that of raw PA6. As previously mentioned, the addition of DIA generated a chain extension reaction and increased the molecular weight and amount of molecular chain entanglement. Consequently, the addition of DIA increased the modified PA6 samples’ elasticity. According to the results of DSC measurements, the crystallinity of PA6 samples reduced after chain extension, which was beneficial to elongation.

In Figure 8b, representative stress–strain curves of different PA6 samples are reported. It can be concluded that strain at break increases with the DIA content, going from 25% for raw PA6 to 59% for the 8 wt% PA6 sample.

## 4. Conclusions

In summary, PA6 foams modified with α-olefin–maleic anhydride copolymer were prepared by melt blending and chemical injection foaming. In GPC testing, the addition of DIA showed the ability to improve the molecular weight of PA6 samples and enhance their rheological properties, including complex viscosity η*, storage modulus G’, loss modulus G”, and loss tangent tanδ for high foamability. Based on DSC and XRD analyses, the crystallinity of modified PA6 samples decreased, the double melting peaks became one peak, and the α crystallites at 20.3° gradually disappeared with increasing DIA content. This could be attributed to the effect of chain extension on crystallization behavior. The addition of DIA effectively increased the water CA and reduced water absorption, which were also useful for foaming ability. In tensile testing, the elongation at break for modified PA6 samples improved due to the use of DIA. Moreover, with the addition of DIA, a smaller cell size and more uniform foam morphology were obtained, the cell density increased, and the cell size distribution became narrow, especially when the amount of DIA added was 5 wt%.

## Figures and Tables

**Table 1:** The Mn, Mw, Mp, and PDI of raw PA6, PA6–DIA2%, and PA6–DIA5% samples.

Sample	Mn (g/mol)	Mw (g/mol)	Mp (g/mol)	PDI
Raw PA6	61,869	131,064	93,713	2.118
PA6–DIA2%	61,303	274,100	135,869	4.471
PA6–DIA5%	8909	247,413	209,476	27.77

**Table 2:** Thermal properties of different PA6 samples.

Samples	Raw PA6	PA6–DIA1%	PA6–DIA2%	PA6–DIA3%	PA6–DIA5%	PA6–DIA8%
T_m1_, T_m2_ (°C)	222.5, 218	222, 218	222, 218	221, 218	219	218
T_c_ (°C)	195	195.5	195.4	195	194	194
X_c_ (%)	13	10	9	7	4	4

## Data Availability

Not applicable.
